# Issues in Grounded Cognition and How to Solve Them – the Minimalist Account

**DOI:** 10.5334/joc.444

**Published:** 2025-04-21

**Authors:** Jannis Friedrich, Martin H. Fischer, Markus Raab

**Affiliations:** 1 Institute of Psychology, German Sport University Cologne, Koln, DE; 2 Potsdam Embodied Cognition Group, Psychology Department, University of Potsdam, Potsdam OT Golm, DE

**Keywords:** Embodied Cognition, Semantics, Action and Perception, Emotion and Cognition

## Abstract

The field of grounded cognition is concerned with how concepts are represented by re-activation of the bodily modalities. Considerable empirical work supports this core tenet, but the field is rife with meta-theoretical issues which prevent meaningfully progressing beyond this. We describe these issues and provide a solution: an overarching theoretical framework. The two most commonly cited grounded cognition theories are *perceptual symbol systems* and *conceptual metaphor theory*. Under perceptual symbol systems, concepts are represented by integrating fragments of multi-modal percepts in a simulator. Conceptual metaphor theory involves a limited number of image schemas, primitive structural regularities extracted from interaction with the environment, undergoing a limited number of transformations into a concept. Both theories constitute important developments to understanding mental representations, yet we argue that they currently impede progress because they are prematurely elaborate. This forces them to rely on overly specific assumptions, which generates a lack of conceptual clarity and unsystematic testing of empirical work. Our *minimalist account* takes grounded cognition ‘back to basics’ with a common-denominator framework supported by converging evidence from other fields. It postulates that concepts are represented by simulation, re-activating mental states that were active when experiencing this concept, and by metaphoric mapping, when concrete representations are sourced to represent abstract concepts. This enables incremental theory development without uncertain assumptions because it allows for descriptive research while nonetheless enabling falsification of theories. Our proposal provides the tools to resolve meta-theoretical issues and encourages a research program that integrates grounded cognition into the cognitive sciences.

## 1. Introduction

Much research and theorizing has been directed at explaining the format of mental representations. The classical approach (cf. Ramsey, 2007) states that cognition involves representations that have the format of amodal symbols, just as words in language are amodal symbols that represent a referent. Yet, over the past four decades, a large body of empirical evidence and theoretical arguments from across the cognitive sciences have amassed evidence to the contrary, that concepts must be grounded in sensory and motor experiences in order to have meaning (Barsalou, 1999; Fischer & Coello, 2016; Shapiro, 2019). Yet, research has reached an impasse, and there has been only limited theoretical progress in uncovering further details regarding the format of mental representations. Here we describe the impasse and how to surmount it.

In this paper we argue that the field of grounded cognition suffers from two meta-theoretical issues which obstruct progress. Both result from prematurely attempting to formulate sophisticated theories without having identified basic principles, which causes both a lack of conceptual clarity and unsystematic empirical work. In this paper we explicate these two issues, and consequently formulate our proposed solution, which we call the minimalist account of grounded cognition. We propose to strip existing theories of their unjustified sophistication, revert back to basics, and progress incrementally on solid footing. To enable this progress, the minimalist account identifies common ground across grounded cognition theories and serves as an overarching theoretical framework. This provides a set of common assumptions and mechanisms, within which opposing theories can pit their respective hypotheses against one another. The minimalist account will improve the value of past and future empirical work, allow for descriptive research, and stimulate a coherent grounded cognition research program.

The meta-theoretical issues described throughout this paper are certainly not unique to grounded cognition, and are pervasive throughout psychology and cognitive science, as a result of fundamental differences in subject matter of ‘harder’ (e.g., mathematics, chemistry) versus ‘softer’ sciences (e.g., psychology, sociology; cf. Fanelli & Glänzel, 2013). Lykken (1991) argues that the harder natural sciences benefit from relative ignorance, which forces them to make *incremental* progress. An example is the model of the atom by J.J. Thomson, which won him a Nobel prize in 1907. This model accounted for the space of known phenomena at the time, yet with a series of experiments from 1909–1911, Rutherford expanded this space. The next model, Rutherford’s own, then improved upon Thomson’s by specifying it (Simonyi, 2012). This example demonstrates the iterative nature of harder sciences: new phenomena reveal issues in existing theories, which in turn become more specific, until new phenomena put these into question. Such iteration is unfortunately not the case in the softer sciences, where the space of phenomena (e.g., intergroup conflicts, emotions) is vast already before the first empirical investigation (Lykken, 1991).

Instead of small incremental steps during which phenomena are first viewed as simplified silhouettes, with theoretical progress consisting of ‘zooming in’ to the details, soft sciences start with the details, being forced to account for the full complex web of causes. Applied to grounded cognition, this meant attempting to explain the detailed mechanics of the human conceptual system (cf. Barsalou, 1999; Lakoff & Johnson, 1980), at a time when the state of knowledge was not able to account for the vast underlying complexity. This explanatory gap forced grounded cognition theoreticians into taking a theoretical leap, making assumptions that were perhaps unjustified. This strategy produced overly detailed theories based on insufficient prior evidence to justify such specificity, and closed the field off to descriptive and explorative research. Below, we outline the consequences of this over-ambitious leap and then our proposed treatment.

We preface our main argument with three remarks. First, we want to emphasize at the outset that the (amicable) criticisms levelled against existing theories will be based on meta-theoretical arguments, not on empirical evidence. We do not attempt to weigh up evidence or declare any theory as validated; rather we argue that the theories’ assumptions were unjustified, which has resulted in a state of research which obstructs theoretical and empirical development. Similarly, we do not assert to know which theories are sufficiently validated, but propose that the best way to identify these is by adopting a simplified overarching theoretical framework as the point of departure for research, the approach we portray here. Therefore, we only intend to shape, not refute, the rich field of grounded cognition to produce a useful tool for future research. It is even possible that central parts of our minimalist account turn out to be incorrect and should be discarded. Although we believe this is unlikely, even such a scenario would be a success for the minimalist account because it would constitute falsification and thereby progress. As we will argue below, falsifying core tenets of current grounded cognition theories is not facilitated by the currently available theories. Therefore, this account intends to be useful and representative of the core mechanisms which past research has elucidated. An empirically motivated assessment of the minimalist account would be important and we invite this below, but implementing it goes beyond the scope of the current paper. We therefore do not intend to minimize the great achievements and valuable contributions of these theories. Indeed, some of their constructs, over 25 years old, are still pertinent and make up our minimalist account, a testament to their visionary nature. Our argument aims to be a contribution to the important research field these theories conceived, and we are fully aware of our debt to their work.

Second, our proposed treatment, the minimalist account, benefits more than just our theories of mental representations. To understand cognition generally, understanding the substrate of cognition is critical. This can be gleaned from the various fields in which work has built on the two central grounded cognition theories discussed here, *perceptual symbol systems* and *conceptual metaphor theory*. Both theories are foundational in research on cognition, including models of memory, perception, and language. Beyond, their widespread influence is demonstrated by the fact that they have even been successfully adopted in the fields of consumer psychology (Landau et al., 2010; Wyer Jr, 2018), psychopathology (Gjelsvik et al., 2018; Törneke, 2020), robotics (Kim & Maher, 2020; Taniguchi et al., 2016), and more (cf. Glenberg, 2010). Yet, their benefit to cognitive science does not emerge just from the minimalist account’s contribution to grounded cognition. Our account also allows to align previously disparate theories, which generates synergies by using the findings from one field to inform another. Scientific progress crucially depends on theory integration (cf. Kitcher, 1995), which such an overarching theoretical framework facilitates and encourages (Muthukrishna & Henrich, 2019). There are, for example, many theories that rely on simulation with which grounded cognition could be aligned (Pezzulo, Candidi, et al., 2013), or theories of memory that similarly give action or perception a constitutional role, such as the Act-In Theory (Versace et al., 2014). Therefore, the account of mental representations and processes provided here, guided by philosophy of science, arrives at a solution for meta-theoretical issues which will benefit and integrate disciplines within and outside grounded cognition.

Third, it is important to mention that we discuss these issues and their solutions in the context of researchers’ behavior. One of our main points is that descriptive work should be emphasized over confirmatory research. Yet, descriptive work is often wrongly considered inferior or less scientific, and therefore less attractive to scientific journals and funding bodies (Braganza, 2020; Haig, 2013; Rozin, 2001). Therefore, the below-listed issues certainly stem in part from the incentives set by these stakeholders, and researchers’ dependency on them. Our proposal should therefore be seen in line with other recent work calling for cognitive and psychological science to embrace bottom-up, explorative, or descriptive research (e.g., Eronen & Bringmann, 2021; cf. also Maner, 2014). We will not focus on the important role of these and other contextual circumstances, instead focusing on issues and solutions internal to the field.

We begin with an overview of grounded cognition and its rationale, followed by descriptions of the two main theories in grounded cognition, perceptual symbol systems and conceptual metaphor theory. We are necessarily limiting the scope of their description to those parts which are necessary for appreciating current conceptual problems.^1^ We then flag the two main issues we identify in grounded cognition: a lack of conceptual clarity and unsystematic empirical work. Having elucidated these issues, we propose our solution: the minimalist account of grounded cognition. It provides a silhouetted view on concept grounding that captures those components of which we are confident, but is not forced to speculate on details. These details are left to future research, which should build on the minimalist account in small incremental steps, as opposed to premature speculative leaps.

## 2. Classic Theories of Grounded Cognition

Grounded cognition is the research program concerned with understanding how mental representations of concepts gain meaning by their embedding in the modalities (Barsalou, 2008; Borghi et al., 2017; Glenberg & Gallese, 2012; Kiefer & Pulvermüller, 2012). This field falls within the broader domain of ‘embodied cognition research’, a term for research characterized by its focus on the body, environment, and situation as a critical component of cognition (Fischer & Coello, 2016; Shapiro, 2007).^2^ It contains various different foci with grounded cognition’s focus on the mental representation of concepts only being one aspect (Clark, 2008; Newen et al., 2018; Wilson, 2002).^3^ Importantly, grounded cognition does not assume that concepts can only be grounded in the body, as may be implied by the term ‘embodied cognition’ (Barsalou, 2020; Fischer, 2024). Indeed, concept grounding can be accomplished by factors beyond the body, which makes the term embodied cognition ill-fitting. It has been proposed that the grounding of concepts is shaped in three ways: Via its situatedness (situation-specific factors like the orientation of one’s head), embodiedness (body-specific factors like being right- or left-handed), and tropism (environmental regularities such as the uni-directional downward pull of gravity; Fischer, 2012; Friedrich et al., 2024; Myachykov et al., 2014; Pezzulo, Barsalou, et al., 2013).^4^

Embodied cognition, and grounded cognition especially, existed first as an opponent to standard cognitive science (Ramsey, 2007). This approach proposed concepts to be amodal symbols, represented much like words in a ‘language of thought’ (Fodor, 1983; Pylyshyn, 1984; Quilty-Dunn et al., 2022). Amodal symbols are a format of mental representation in which the representation is not in a sensory or perceptual format. They are therefore often arbitrary, sharing no features with their referents. Words, for example, usually share no features with the referents they represent (e.g., the word lamp does not look like a lamp), and are instances of arbitrary symbols. This stands in contrast to the modal representations of grounded cognition, which represent concepts by sensory and motor re-activations resembling those present when interacting with the conceptual referents. Amodal-symbolic theories of cognition gained significant popularity in the cognitive revolution. Yet, the strength of this hypothesis was called into question, in part based on the ‘symbol grounding problem’ (Harnad, 1990; Searle, 1980; Steels, 2008). The symbol grounding problem points out that amodal symbols could not bear meaning if they were not attached to physical experiences, and that an amodal approach to mental representation fails on this account.

Today, the symbol grounding problem is best demonstrated by looking to large-language models like ChatGPT. There is an intuitively accessible qualitative difference between the ‘cognition’ of large-language models and human cognition. Cognition in large-language models is the result of training a mathematical model on a corpus of language which allows the model to identify what words are likely to follow others. This allows to generate responses which contain information and seem intelligent, yet are in effect based on sophisticated pattern completion. In this sense, a large-language model does not know the meaning of its answers because each word only refers to other words. It may say that the apple is red, but only because it knows that apple is often mentioned in the context of red (alongside tree, sweet, and pie). Yet it cannot be said that the model truly knows what red means because the ‘meaning’ of red only consists of its co-occurrence with these and other words, e.g., sunrise, wine or communist. Put differently, large-language models’ symbols are not grounded. Their concepts are encapsulated because their meaning derives solely from their co-occurrence statistics to other words, and they cannot have experiences (Abbate, 2023; Pezzulo, Parr, et al., 2024). We humans, on the other hand, have experiences, and our mental representations are *about* the external world. This aboutness grounds our cognition, because it grants mental representations their content (Pezzulo & Castelfranchi, 2007), such that the color of an apple is the experience of red, and not ‘somewhere between apple, communist, sunrise, and wine’. This issue of large-language models’ symbols failing to bear meaning directly translates to amodal theories of concept representation, which similarly suffer from not being connected to experience. Grounded cognition solves the symbol grounding problem by stating that symbols are modal, and grounded by virtue of consisting of a re-activation of modal experiences. This means that the apple is represented by re-activating the sight of redness, the sweet taste or the sound of a crunch when one bites into it. This is supported by imaging evidence that both sensory and motor areas in the brain activate during lexical access (e.g., Kuhnke et al., 2020). For example, in order to understand the concept kick, similar brain areas are active as when performing the action itself (Hauk et al., 2004; Pulvermüller & Fadiga, 2010).

Perhaps the most central developments in grounded cognition research have been related to the kinds of experiences within which concepts can be grounded. In early work, this was often argued to be the sensorimotor system (Barsalou, 2008). One issue which confronted such theories, though, was that of abstract concepts. Compared to concrete concepts (e.g., cup or mountain), which are experienced by exteroceptive modalities, abstract concepts (e.g., justice or democracy) are by definition intangible, and therefore could hardly be represented by the sensorimotor system (Barsalou, 2003a; Dove, 2009; Mahon & Caramazza, 2008). Yet, this critique failed to account for the diversity of experiences: Abstract concepts can be grounded in interoception and meta-cognitive states (e.g., the experience of recognizing something as true; Barsalou & Wiemer-Hastings, 2005; Connell et al., 2018; Prinz, 2012), as well as emotions (Kousta et al., 2011; Vigliocco et al., 2009). Later, the role of language as a means of representing concepts was also emphasized (Dove, 2011; Louwerse, 2011), as well as contributions from the social world (Borghi & Cimatti, 2009; Reinboth & Farkaš, 2022), and the role of predictive processing (Borghi et al., 2019; Van Elk & Bekkering, 2018). Today, a ‘hybrid’ view is most widely accepted, acknowledging that concepts are grounded in the aforementioned substrates, but also in ‘amodal’ formats (Dove, 2011; Fischer & Shaki, 2018). Hybrid theories tend to acknowledge that both representational formats exist and, depending on the cognitive task or situation, are selectively recruited (e.g., Dove, 2014; Paivio, 1990; Patterson et al., 2007; Zwaan, 2016).

Yet, these grounding substrates do little more than correlate a brain state to a behavior. The more interesting question is *how* these experiential aspects of cognition come to represent concepts. There are many approaches and theories in grounded cognition (see Table 1). Based on the names used in publications, it is not always clear to which theory an author is referring, and to what degree they subscribe to details of the theory. We have aligned these as best as possible in the table below, but do acknowledge that this merely flags up the current lack of clarity in concept use in grounded cognition. We now discuss the two most central theories in turn, which we believe cover the vast majority of theoretical positions in the literature on grounding concepts (including the concepts purported by other theories, cf. action schemas below), and which are most frequently cited by experiments and reviews (see Table 1). Then we identify two key epistemological issues within the field. This leads us to the minimalist account of grounded cognition as an overarching theoretical framework, which provides a common basis and solution to the issues.

**Table 1:** Overview of Grounded Cognition Theories as Named in a Representative Sample of Literature Reviews.


SOURCE PAPER AND THEORY NAMES	THEORY NAMES IN THE PRESENT PAPER

**Glenberg et al., (2008)**	

Metaphor Simulation Action Schemas	Conceptual Metaphor Theory Perceptual Symbol Systems N/A (uses metaphor)

**Barsalou (2008)**	

Cognitive linguistics Theories Theories of Situated Action Cognitive Simulation Theories (perceptual symbol systems) Cognitive Simulation Theories (memory theories) Cognitive Simulation Theories (social simulation)	Conceptual Metaphor Theory N/A Perceptual Symbol Systems N/A N/A

**Anderson (2008)**	

Glenberg & Kaschak Simulation Conceptual metaphor	N/A (uses metaphor) Perceptual Symbol Systems Conceptual Metaphor Theory

**Dove (2011)**	

Metaphoric extension Action Schemas Situated Simulation	Conceptual Metaphor Theory N/A (uses metaphor) Perceptual Symbol Systems

**Borghi et al. (2017)**	

Action Schemas Force Dynamics Conceptual Metaphor Theory Introspective and situational Properties Affective embodiment Account Language as Situated Simulation Dove’s representational pluralism	N/A (uses metaphor) N/A (uses metaphor) Conceptual Metaphor Theory N/A (in Perceptual Symbol Systems) N/A Perceptual Symbol Systems N/A

**Pexman (2019)**	

Conceptual Metaphors Language Emotion	Conceptual Metaphor Theory N/A N/A

**Pecher & Zeelenberg (2018)**	

Conceptual Metaphor Theory Situated Cognition Hybrid Models	Conceptual Metaphor Theory Perceptual Symbol Systems N/A

**Fincher-Kiefer (2019)**	

Glenberg’s action schemas Lakoff & Johnson’s Metaphors Barsalou’s Situated Simulation	N/A (uses metaphor) Conceptual Metaphor Theory Perceptual Symbol Systems

**Friedrich et al. (2024)**	

Simulation Conceptual Metaphor Theory Conceptual Spaces Predictive Processing	Perceptual Symbol Systems Conceptual Metaphor Theory N/A N/A


*Note*. These are select papers that review grounded cognition and the name they chose for the theories they assessed. We do not intend for this to be a comprehensive listing of all reviews, and limit it to reviews that focused mostly on embodied theories (as opposed to weak or un-embodied theories, cf. e.g., Meteyard et al., 2012, Muraki et al., 2023). Nonetheless, we argue these reviews are representative of the use of nomenclature in grounded cognition research.

### 2.1. Perceptual symbol systems

*Perceptual symbol systems* as a theory states that concepts are represented by multimodal re-enactments of percepts involved when experiencing those concepts (Barsalou, 2008, 2020).^5^ It argues that fragments of multimodal perceptions are isolated to become represented as perceptual symbols (Barsalou, 1999). When later representing the concept, these perceptual symbols are recombined into so-called simulators that are equivalent with concepts and form the basis of all cognition. For example, when someone mentions that “there is a cup on the table”, various contextually-relevant perceptual symbols (the shape of a table or the color of a cup) are activated in a simulator, which constitutes the mental representation. This holds even for abstract concepts which differ from concrete concepts by their grounding substrate: Concrete concepts mostly involve simulations of perceptual symbols from the modalities of the sensorimotor system, while abstract concepts involve simulations of interoception, or meta-cognitive states (Barsalou & Wiemer-Hastings, 2005; Kiefer & Barsalou, 2013). For example, in order to verify the concept of truth, the simulation of that coffee cup on a table can be mapped to the visual perception of seeing it, and the meta-cognitive appraisal of successfully matching the two becomes the abstract concept of truth. Perceptual symbol systems emphasizes the importance of interoception and meta-cognitive states, but this is not to say that other theories do not also purport grounding via these modalities.

The details of this theory, and how it is applied, differ depending on the authors and the foci of their respective research. For example, it has been differently named with different emphases. *Situated conceptualization* argues that simulators are rich context-dependent representations, often with a focus on emotion (Lebois et al., 2020; Wilson-Mendenhall et al., 2011). The perspective effect, where the perspective from which a scenario is described affects what attributes of an object become particularly salient, also supports this view (Borghi et al., 2004; Borghi & Barsalou, 2021). Another example includes modality-switch costs, which reflect concepts described in one modality inhibiting concepts associated with another modality (Pecher et al., 2003). This view was extended further to become the *language as situated simulation* hypothesis (Barsalou et al., 2008). It emphasizes more than its predecessors the role of language as a shortcut or pointer to concepts. This understanding of cognition as experiential simulation connects to the larger *situated action cycle* which describes how such modal simulations connect to action (Barsalou, 2020, 2021).

There is an important difference between the *perceptual symbol systems* theory and the mechanism of *simulation*, despite them often being equated. The theory brings with it the constructs and processes posited in seminal theory papers (Barsalou, 1999, 2008), including simulators which are equivalent to a concept and convergence zones, which enable abstraction (Simmons & Barsalou, 2003). Yet when other work references this theory, it typically intends to signify allegiance to the single *mechanism*, not the *theory*. The mechanism of simulation only reflects the covert re-activation of modal perceptions (Pezzulo, Candidi, et al., 2013; Reilly et al., 2023), without the added theoretical baggage, like simulators or supramodal convergence zones (although, indeed, abstraction will be an important ability for future research to explain). This distinction will become important when we propose our minimalist account below.

### 2.2. Conceptual metaphor theory

In contrast to perceptual symbol systems, *conceptual metaphor theory* states that concepts are represented by re-using representations from other, more concrete, domains to represent a target domain (Gibbs, 2005a; Kövecses, 2020a; Lakoff & Johnson, 1999). More specifically, it argues that throughout human experience primitive schemas (e.g., CONTAINER, which originates from the many instances of bottles, cups, or boxes holding other objects) repeat and become cognitively represented in the form of structured patterns (Johnson, 2018). These patterns, called *image schemas*, are transformed in a limited number of ways, such as by the ‘multiplex-to-mass’ or ‘following-a-trajectory’ transformations (Gibbs & Colston, 1995). These transformations turn image schemas into *conceptual metaphors* (Johnson, 1987; Lakoff, 1987; Langacker, 1986; Talmy, 1983) which constitute the mental representation of the concept. An example for this chain of transformations is the image schema of SOURCE-PATH-GOAL which is applied to the target domain of love, to become the conceptual metaphor LOVE-IS-A-JOURNEY (which is the origin for sayings like “look how far we’ve come” or “we have reached the end”; Gibbs, 2005a, p. 92). This then also elicits embodiment signatures in behavior (Gibbs, 2006). For example, the abstract concept of time is understood by redeploying the more concrete domain of space, such that forward movement in space leads to a change in the representation of the abstract concept time, toward the future (Boroditsky, 2000; Loeffler et al., 2017). In short, conceptual metaphor theory argues that, via image schemas, all conceptual processing is reasoning that is either image-based (Lakoff, 1990) or “visual, auditory, kinesthetic, and tactile” (Gibbs & Colston, 1995, p. 394). Primary metaphors are also relevant, although their relation to the other constructs is not fully clear (Grady, 1997, 2005) and they do not appear in all texts describing conceptual metaphor theory (e.g., B. Winter & Matlock, 2017). A more recent development of this theory argues for different levels in the hierarchy of representation, consisting of the image schema at the most abstract level, followed by the *domain*, the *frame*, and the *mental space* as the least abstract level (Kövecses, 2017, 2020a).

As in perceptual symbol systems theory, reference to conceptual metaphor theory often does not signify allegiance to the full theory, but rather to its core mechanism, which in this case is *metaphoric mapping* (see Table 1). The full theory involves image schemas, transformations, and primary metaphors. The mechanism of metaphor, and the way it is most frequently referenced, merely describes that an abstract domain is represented by re-deploying representations from a concrete domain (Casasanto & Gijssels, 2015; Jamrozik et al., 2016). This view of metaphor relates to the *action schema* approach (Glenberg et al., 2008), a theory often posited to be distinct from conceptual metaphor theory (see Table 1). A prototypical example from this theory is the work of Glenberg and Kaschak (2002) who showed that linguistic descriptions of object transfer along the midsagittal plane (toward or away) influence response time when responding with movements along the same axis. Importantly, in relation to metaphoric mapping, this effect holds also for abstract descriptions like “she told you the story”. This action sentence compatibility effect (ACE) suggests that meaning comprehension is related to action planning and that comprehension of even abstract concepts relies on action schemas. The action schema approach, despite relying on the same mechanism as conceptual metaphor theory is treated as separate, and this repetition is left unaddressed.

## 3. Issues in Grounded Cognition

We argue that characteristics of the theories and their consequent application obstruct progress in understanding the grounding of mental representations. We begin this section by describing the current state of gridlock in grounded cognition research, its historical background, and detail the two responsible issues (see also Table 2): a) a lack of conceptual clarity, and b) unsystematic empirical work.^6^

**Table 2:** Issues in Grounded Cognition Research and Examples.


LACK OF CONCEPTUAL CLARITY	
Conceptual Clutter	Conceptual Metaphor Theory (Lakoff, 1987), Metaphoric structuring (Boroditsky, 2000), Neural theory of language (Gallese & Lakoff, 2005), ‘Extended’ conceptual metaphor theory (Kövecses, 2020)
Jingle-Jangle	*Jingle*: Simulation (Barsalou, 2008; Battaglia et al., 2013; Gallese, 2005; Shanton & Goldman, 2010; Hesslow, 2012) *Jangle*: Schema structure (Lakoff, 2014) = Cognitive primitives (Lakoff, 2012) = Image schemas (Lakoff, 1987)

**UNSYSTEMATIC EMPIRICAL WORK**	
No Falsification	
Lack of theoretical allegiance	*No Allegiance*: e.g., Dils & Boroditsky, 2010 *Smudged Allegiance*: e.g., “Embodied Cognition Approach” (Fischer & Brugger, 2011, p. 5; Tolentino-Castro & Raab, 2021, p. 1)
Cherry-picking theoretical support	ACE cited for perceptual symbol systems, action schemas, conceptual metaphor theory (cf. Anderson, 2008)
No descriptive research	How frequent is metaphor use? What are all possible grounding substrates? How many image schemas exist?


The state of theoretical knowledge in grounded cognition research has not significantly developed in recent years (Zwaan, 2021). The most cited theories are still the aforementioned two (as also evidenced by Table 1), and when these are cited, the respective original texts are referenced, which date back 26 and 45 years, respectively (Barsalou, 1999; Lakoff & Johnson, 1980). This suggests that there are few important changes or refutations which would make the respective references outdated. Newer accounts, such as the Words as Social Tools, the action schemas approach, or the affective embodiment account (Borghi & Cimatti, 2009; Glenberg et al., 2008; Kousta et al., 2011), do not refute or build on these prior theories, and are also by now over 10 years old. In their expansive review, which aimed to collect all accounts of grounding abstract concepts, Borghi et al. (2017) mention eight, mostly incompatible, theories of which none were conceived later than 2010. These examples together demonstrate that theories are put forth but consequent research fails to meaningfully build on them. This theoretical impasse is further indicated by the fact that descriptions of the grounded cognition approach in very recent papers do not differ substantially from those published ten or fifteen years prior; They are consistently limited in their theoretical detail to vague descriptions, with the core content being that ‘concept representations involve the reactivation of modality-specific representations from sensorimotor systems’ (e.g., Kemmerer, 2022; Michel, 2023). There is therefore, despite much time and empirical effort being invested, little additional understanding in grounded cognition, in the past ten or perhaps even twenty years. What is the cause of this gridlock?

We argue that the present state of gridlock in progressing the understanding of the format of mental representations originates from these theories having attempted to advance prematurely (cf. Bringmann et al., 2022): Wide-sweeping grand claims postulated specific mechanisms, when the incremental first steps towards establishing such mechanisms had not been convincingly completed. The first evident consequence of prematurely elaborate theories is that a theory likely misses the mark when it extrapolates too far beyond the limit of the current state of knowledge. For example, consider conceptual metaphor theory’s hypothesis that image schemas undergo one of a limited number of transformations to become conceptual metaphors, which in turn serve to represent concepts. With little empirical evidence for the cognitive reality of image schemas, the subsequent steps become increasingly unlikely. A research program building on this, and similar fastidious hypotheses and overparticular constructs, will be unlikely to make accurate predictions (Trafimow & Earp, 2016). Therefore, our first assessment is that theories which attempt to be too elaborate too early are more likely to lead down false and unproductive paths of research (Bringmann et al., 2022). An example of work which did not fall prey to this mistake comes from numerical cognition, where the initially rather vague postulate of a “quasi-spatial” mental number line (Dehaene et al., 1993, p. 394) inspired a wealth of specifying research such as identifying its metrical and dimensional properties (Fischer & Shaki, 2014; B. Winter et al., 2015).

Yet, over-specificity alone would not constitute a severe meta-theoretical issue: An incorrect theory should, in principle, be falsifiable and thus promptly discarded (cf. Popper, 1959), yet the current state of grounded cognition is not amenable to such falsification. In an ideal world, with two opposing theories, the next step would be theory-testing via falsification: identifying critical hypotheses to find the theory that better^7^ accounts for experimental findings (Platt, 1964; Popper, 1959). At first this may seem simple because obvious and important discrepancies exist between them, like: Do image schemas exist? If so, how many? Do simulators exist? These specific questions, too numerous to list in full, already point to a problem: the theories do not share a common ground. Their most fundamental positions (e.g., what is the most basic building block of cognition, image schemas or fragments of perception?) differ already, obstructing systematic empirical assessment. In other words, perceptual symbol systems and conceptual metaphor theory make strong assumptions which prevent comparisons. Furthermore, with such ostensibly sophisticated theories, empirical research that is focused on describing or exploring such basic phenomena does not have a theoretical home in which to embed its findings.

Next, within these overly specific theories, as a result of different authors disagreeing about different assumptions (e.g., whether image schemas, primary metaphors, or mental space are the most basic building blocks of cognition), different versions of overly elaborate theories exist, and this leads to two problematic trends which we will now characterize in more detail. First, a lack of conceptual clarity comes about, reflected in terminological confusion and inflation. Such a lack of conceptual clarity subsequently results in unsystematic empirical work that engages in neither falsification nor descriptive research. This is either due to being forced to remain theory-neutral in order to avoid buying into unnecessary theoretical baggage, or due to designing and operationalizing experiments which must make strong implicit assumptions (e.g., a limited number of image schema transformations; see Table 2).

### 3.1. Lack of Conceptual Clarity

Much progress in science revolves around *commensurability* of theories: when two theories address the same phenomenon, they can be integrated to inform each other and gradually expand to explain novel observations (Kuhn, 1962; Oberheim & Hoyningen-Huene, 2009). *Incommensurability*, on the other hand, describes a state in which theories cannot be combined, threatening progress. Incommensurability is often the result of a lack of conceptual clarity (Hagger, 2014) because theories that use different terms to address the same phenomena cannot be integrated. This often results from *conceptual clutter* (Jones et al., 2016; Morrison & Grammer, 2016) and related, the *jingle-jangle fallacy* (Block, 1995; Kelley, 1927), where similar terms for different constructs (jingle) and different terms for the same construct (jangle) co-exist (Dang et al., 2020; Wulff & Mata, 2023). Conceptual clutter prevents the cumulative progress of science because theories cannot converge in order to build on one another (Gerring, 1999; Locke, 2003).

Conceptual clutter is found throughout grounded cognition theories, highlighting the need for commensurate constructs. For example, conceptual metaphor theory proposes the existence of image schemas (also called “cognitive primitives” in Lakoff, 2012, or “schema structure” in Lakoff, 2014) and primary metaphors. In a coherent theoretical framework, these two components should be co-occurrent, yet many important works exist where only image schemas, and not primary metaphors, are mentioned (e.g., Gibbs, 2005b; Richardson et al., 2003). Alternatively, sometimes only primary metaphors are discussed (Lakoff, 2014; Winter & Matlock, 2017; although Lakoff uses the term “primitive metaphor”, which may be the same as cognitive primitives, another name for image schemas; Lakoff, 2012), or image schemas and primary metaphors as taking place on the same level (e.g., Kövecses, 2020b). Furthermore, beyond image schemas and primary metaphors, a wealth of other categories exists. Some authors distinguish structural, orientational, and ontological metaphors (e.g., Łącka-Badura, 2016; Lakoff & Johnson, 1980), or spatial relation- and force-type ‘cogs’ (another term for cognitive primitives, i.e., image schemas; Lakoff, 2012; also attributional vs. relational metaphors; Gentner et al., 2001; or resemblance vs. correlation-based metaphors; Grady, 2008). Even when these terms are mentioned alongside one another, their functions within theories are not consistent: Some authors state that image schemas are the basic building blocks (Kövecses, 2008), others focus on primary metaphors (Grady, 1997; Lakoff, 2014). Without this consistency in their function, these terms become useless as a reference. A similar problem arises from the absence of an accepted or widely used definition of image schemas (Pecher et al., 2011). They are at times described as kinetic analog representations of spatial relations (Gibbs & Colston, 1995), experiential (Gibbs, 2005b) or schematic (Kimmel, 2009) gestalts, preconceptual structures, etc. (cf. Hampe, 2005; Mandler & Pagán Cánovas, 2014). It is also unclear how many there are, ranging from around two dozen (Johnson, 1987; Lakoff, 1987) to hundreds (Cienki, 1997; Johnson, 2018).

Across grounded cognition we find jingle and jangle in constructs, constituting many slightly-different versions of the same theory, thereby preventing continuity between and within theories. The various names given to grounded cognition theories are examples of jangle (for an overview see Table 1), being very similar but different-sounding. In conceptual metaphor theory some theoretical enclaves (new versions of theories which differ slightly from the original but nonetheless rely on many of the same central posits) are Lakoff’s (1987) work, Boroditsky’s *metaphoric structuring* account (Boroditsky, 2000), the *neural theory of language* (Gallese & Lakoff, 2005), a novel *extended conceptual metaphor theory* (Kövecses, 2020a), or just *metaphor theory* (Merritt et al., 2010). Similar jangle exists in perceptual symbol systems theory which has been called: perceptual symbol systems (Barsalou, 1999) or perceptual symbol systems theory (Horchak et al., 2014), situated conceptualization (Barsalou, 2009), situated simulation (Barsalou, 2003b), language as situated simulation (Barsalou et al., 2008), or often just simulation (Glenberg et al., 2008). These are, of course, not to be confused with the ‘embodied simulation theory’ (Gallese, 2005; Gallese & Sinigaglia, 2011) or the many other different names for ‘simulation’ theories (Pezzulo, Candidi, et al., 2013), which presents an instance of jingle.

These examples of conceptual clutter and jingle-jangle are symptoms of premature advancement, and generate obstacles to meaningful theoretical progress. Firstly, theories that attempt to be elaborate and detailed, without adequately understanding their most fundamental components, result in many enclaves and disputes about details of constructs that are in themselves speculative. Second, prematurely specific theorizing closes the field off to valuable descriptive and exploratory (as opposed to ever more theory-laden) research, which only exacerbates the development of theoretical enclaves. This triggers a cycle in which development is impeded because the theories are not constrained by basic phenomena, which cannot be discovered as a result of little descriptive research, which is in turn the result of conceptual clutter, which prevents discovering basic phenomena to constrain theories, and so on. Together, these trends all prevent pruning of unsupported constructs. The resulting impediment of theoretical progress is evident. If even within conceptual metaphor theory there are many different versions of a theory, which each only permits tests within itself, how should this form a coherent whole to contest against perceptual symbol systems theory?

Philosophers of science have proposed different solutions for a lack of conceptual clarity (cf. Podsakoff et al., 2016; Wulff & Mata, 2023), but we argue that grounded cognition is just not yet ready for grand theories which can justifiably posit such specific constructs. Instead, the field should ‘go back to basics’ (Bringmann et al., 2022). We argue that going back to basics consists of reverting to a silhouetted and non-detailed point of departure, and progressing with bottom-up, inductive research (cf. Aguinis & Vandenberg, 2014). This approach ensures that there are relatively few assumptions.

We choose to focus on two basic mechanisms in the following minimalist account, without assuming any further details. This simplification is in contrast to conceptual metaphor theory’s postulating a limited number of image schema transformations, which already takes for granted the existence of image schemas. Its further claim about a limited number of transformations in turn assumes that something innately limits the amount of transformations, and so on. Without making such unfounded assumptions, the minimalist account should be attractive, no matter which theory’s prior assumptions one disagrees with. Moreover, when using the minimalist account, any departure from, or extension to, the theory should be well-evidenced, which should in turn limit the creation of theoretical enclaves. A common overarching theoretical framework such as this prevents conceptual clutter by generating commensurability between camps of grounded cognition, and across cognitive science, therefore encouraging a coherent research program.

### 3.2. Unsystematic Empirical Work

How does empirical work position itself when multiple incommensurable enclaves of theories exist? Some authors have argued it generates “a potpourri of disconnected empirical findings” (Muthukrishna & Henrich, 2019, p. 2), or in other words, unsystematic empirical work. Empirical work in grounded cognition is characterized by two strategies: authors either do not align with one specific theory, preferring to avoid the murky waters of conceptual clutter and theoretical baggage; or they already agree on the theory’s basic assumptions (e.g., the existence of simulators). Both strategies fail to serve the goal of falsification. Perhaps most intuitively, this problem is reflected in the fact that empirical work prefers to reduce overly elaborate theories to their basic mechanisms during operationalization, suggesting that experimenters intuit that this is the boundary of what is currently known.

The lack of rigorous theory-testing is harmful, and yet pervasive in the field, as demonstrated by both experimentally-driven and theoretical work. First, experimental work that is clearly located within grounded cognition, and that also draws on related theoretical and empirical work (as indicated by citations), often does not refer explicitly to either “embodied cognition” or “grounded cognition” at all, let alone to specific theories (e.g., Dils & Boroditsky, 2010; Kaschak et al., 2005). On the other hand, the authors could declare allegiance to a specific theory (e.g., Richardson et al., 2001), but this incurs a full allegiance to all detailed postulates, which is rare. Additionally, because of the strong assumptions which accompany these theories, the outcomes cannot subsequently be compared against another theory. For this reason, common practice is less stringent: In most cases where allegiance is proclaimed, it smudges over the theories within the field. There is reference to so-called “embodied effects” that are theoretically backed by citing vague entities like “theories of embodied cognition” (Jostmann et al., 2009, p. 1169) or the “embodied cognition approach” (Fischer & Brugger, 2011, p. 5; Tolentino-Castro & Raab, 2021, p. 1; Werner et al., 2019, p. 171), which are similarly-vaguely defined by e.g., “thoughts comprise simulations of bodily experience” (Casasanto, 2009, p. 351) or “a bidirectional interplay between cognitive processes and the human body” (Werner et al., 2019, p. 171), followed by a listing of citations including popular works from both perceptual symbol systems and conceptual metaphor representatives of the grounded cognition field. Without allegiance to a specific theory, the empirical work evidently cannot provide strong evidence in support or against it.

Second, theoretical work often involves authors cherry-picking experimental results collected under the name of a different theory. To illustrate, Anderson (2008) underlines three findings that have been deployed to support multiple different, ostensibly contradictory, grounded cognition theories. One of these findings is the seminal ACE (Glenberg & Kaschak, 2002), which has been cited in support of the authors’ own theoretical framework of action schemas, as well as in support of perceptual symbol systems and also its opponent, conceptual metaphor theory (see below for the minimalist account’s parsimonious solution to this). Even work by critics of grounded cognition is chosen as support of grounded cognition when deemed suitable (Anderson, 2008; Ostarek & Bottini, 2021). In other words, the ‘theory-testing’ practices in grounded cognition research often involve theories not being tested, but rather experimental results being selected post-hoc, resulting in confirmation bias.

In contrast to these practices, successful research programs engage in small-step theory-building, followed by systematic testing of predictions deduced from these theories (Kitcher, 1995; Popper, 1959). The field of grounded cognition is currently performing *discovery-oriented* research that is focused on confirming ostensibly surprising ‘embodied cognition effects’, but should move to *theory-testing* research (Oberauer & Lewandowsky, 2019). It has found enough headline results (discoveries) to be taken seriously as standard cognitive science’s antagonist, and should now begin testing its theories by falsification (cf. also Barsalou, 2010; Fischer, 2024; Popper, 1959). It could even go a step further and perform strong-inference testing, pitting two ‘critical’ hypotheses derived from opposing theories against one another (Ostarek & Bottini, 2021; Platt, 1964). To foster engagement in falsification or strong-inference, a grounded cognition account without unnecessary assumptions must be put forward to which experimental work aligns itself comfortably without buying into a host of theoretical addenda. Therefore, this problem too, is counteracted by going back to basics with a framework of reduced detail and agreed-upon mechanisms.

Beyond not serving falsification, prematurely advanced theories are also closed off from descriptive research, which constitutes the foundation of the topic under investigation (Bringmann et al., 2022; Rozin, 2001). In contrast to discovery-oriented research, which has a surprising hypothesis that is based on a theory which it aims to confirm, descriptive work merely involves understanding a phenomenon better, often with few top-down expectations that it aims to confirm. Prematurely advanced theories force exploratively-minded researchers to buy into significant amounts of theoretical detail or into smudging over theoretical allegiance. The problem with examples from empirical research listed above does not rest with their authors. Researchers often perform basic descriptive or explorative work, which is a valuable and necessary endeavor. This holds especially in a field such as grounded cognition which has limited background knowledge of its context and basic foundation, as explicated above. Yet, researchers are rightly hesitant to align with overly elaborate theories for risk of incurring unwanted theoretical baggage (e.g., assuming image schemas buys in transformations and primary metaphors). This leads researchers to instead smudge over the specific theoretical allegiance and instead reference a wide set of theories. Yet, experimental research, whether explorative or not, is required to be situated within the bigger theoretical picture. If not situated in a bigger theoretical picture, as is the case for much research on grounded cognition, empirical work cannot build on one another and each experiment must restart at zero. This is why the premature advancement has stifled progress: For a field like grounded cognition, which may not yet have such expansive foundational knowledge, descriptive and explorative research is critical; prematurely advanced theories are closed off from, and indeed obstruct, such work.

This problem is evident in the relative lack of basic knowledge, and comparatively much discussion, about details of concept grounding. For example, while the complicated process of how image schemas transform into conceptual metaphors has been exhaustively discussed, the basic description of the frequency of metaphor use for various abstract concepts has received comparatively little (none, to our knowledge) attention. Similarly, while there exists dispute on the intricacies of simulators in convergence zones, there have been few explorative assessments of what types of representations can form grounding substrates (see below). This practice carries real effects later in the research cycle.

One example of the detrimental effects of a lack of descriptive knowledge comes from the non-replication of the aforementioned ACE by Morey et al. (2022; see also Papesh, 2015). These authors, among them the original study’s creators, performed a stringent replication and failed to find the effect. Yet, it has since been argued that this non-replication originates from subtle differences in experiment designs, and that a lack of general understanding of grounding is to blame (Teskey et al., 2024). For example, a third variable could be responsible, as suggested by the fact that there is strong inter-individual variability in the size and direction of the effect (A. Winter et al., 2022). Alternatively, minute differences in stimuli might be to blame, because replication is successful when the focus of the sentence is on the action (Jin et al., 2024). This opaqueness is further reflected by authors of the non-replication, who themselves state that their null result does not speak against sensorimotor grounding, but rather that their experiment is not effective in uncovering the effect (Morey et al., 2022). This is a clear signal for a lack of foundational knowledge. In fact, this replication, cites various contrary approaches, but not the approach which ACE is commonly brought in connection with: the action schemas approach (see e.g., Borghi et al., 2017; Dove, 2011; Glenberg et al., 2008). The original studies on the ACE effect cited multiple frameworks, including the indexical hypothesis (Glenberg & Robertson, 1999), which is mostly absent from later work discussing the ACE effect (which frames it as support for the action schemas approach). Research on the ACE effect had no overarching theoretical framework, and therefore studies which struggled to elicit the effect could not be meaningfully connected, producing a potpourri of contextual effects. With more descriptive knowledge about the basic mechanisms, integrated into an overarching theoretical framework, empirical research on the effect could be better informed and reasoned about. This descriptive knowledge could, for example, be the time course of ACE, namely that simulations only reach a detectable threshold after repeating the inductive stimulus for longer than a certain time.^8^

In short, the prematurely advanced theories contribute to preventing a valuable description and characterization of phenomena, thereby leading to the aforementioned potpourri of disconnected empirical findings (Haig, 2013; Muthukrishna & Henrich, 2019), and finally a lack of cumulative progress. For this reason, the field requires an overarching framework which allows descriptive and explorative works to find a home and build on one another.

## 4. Synthesis – The Minimalist Account of Grounded Cognition

Having outlined the two main issues currently obstructing progress in grounded cognition research, we now provide a remedy: the minimalist account of grounded cognition. It is an *overarching theoretical framework* (Muthukrishna & Henrich, 2019), a description more general than one specific theory. Overarching theoretical frameworks intend to be a common denominator across multiple theories, such as Darwin’s evolution by natural selection, which, despite originating as a specific theory, has developed into the larger Darwinian framework. Within this overarching framework multiple theories compete, yet all align with the mechanisms of evolution by natural selection. An overarching theoretical framework is important to ensure the value of research, especially in the softer sciences, which often lack this scaffold (Muthukrishna & Henrich, 2019). In the current case it also specifically targets the severe issues we discussed above.

In order to be viable, the minimalist account should require few assumptions, thereby rendering it epistemologically secure, which should in turn encourage empirical work to align itself with it. We take as inspiration William James’ call to make “the science be as vague as its subject” (James, 1890, p. 6; cf. also Kaup et al., 2024, p. 326) and provide an intentionally vague description. Our silhouetted description of grounded cognition does not aim to explain all details of findings in grounded cognition research. Instead, being unspecific about details allows us to relinquish assumptions, thereby gaining more certainty and freedom from the issues described above.

### 4.1. Exposition of the Minimalist Account

With the field of grounded cognition being defined as understanding how mental representations of concepts gain meaning by their embedding in the modalities (see above), we build on this description by proposing two central mechanisms through which modal activation can represent concepts. In short, the minimalist account consists of taking only the core mechanisms from both of the above main theories of grounded cognition, and discarding the assumption-laden theoretical baggage.^9^ First, our account of grounded cognition simply states that concepts are simulated directly (cf. Barsalou, 2003b; Barsalou et al., 2008). The mechanism of *simulation* posits that a concept is represented by re-activating the mental or sensorimotor states which were active during the experience of the concept (Fischer & Zwaan, 2008; Körner et al., 2023). Second, it states that these simulations can represent other, more abstract concepts. In other words, they can be metaphorically re-used (from conceptual metaphor theory; Jamrozik et al., 2016). The *metaphoric mapping* mechanism involves applying the representation from a concrete source domain to understand an abstract target domain. In other words, what is represented is not the abstract concept itself, but a concrete experience, similar enough to form a sufficiently useful representation of the abstract concept (Casasanto & Gijssels, 2015). This means that, under the minimalist account, concept representations consist of simulations of the experiences of the respective concept, and that abstract concepts are especially likely to involve re-deploying simulations that were previously acquired for concrete concepts. This suggests also a nesting of these concepts, with simulation being universal and metaphoric mapping being a specific type of simulation. Beyond these basic mechanisms, any further constructs will require strong empirical support to justify their inclusion into grounded cognition theorizing.

#### 4.1.1 Simulation

The mechanism of simulation underlies, explicitly or implicitly, nearly all work on grounded cognition and vast swaths beyond. It is defined as the re-activation of states, typically caused by outside referents, in the absence of these referents (Barsalou, 2008; Reilly et al., 2023). These simulations can take the form of sensorimotor, meta-cognitive, social or any other prior experience (Borghi et al., 2019; Pezzulo, Barsalou, et al., 2013; Svensson et al., 2009). Simulation is mentioned in nearly every review of grounded cognition. It is also the mechanism which grounded cognition critically relies on, as the representation of concepts in their absence, under grounded cognition, states that modal representations are re-activated. Further, beyond perceptual symbol systems, which places the strongest focus on simulation, this mechanism exists throughout grounded cognition. Its role in conceptual metaphor theory has been emphasized in past reviews (e.g., Pecher et al., 2011), and conceptual metaphor theory has been integrated with simulation either implicitly (e.g., through nearly identical use of simulation and conceptual metaphor in Gibbs, 2005b, 2011) or explicitly (Johnson, 2018). It could even be argued that conceptual metaphor theory relies on simulating image schemas. To represent conceptual metaphors, image schemas are activated in absence of their concrete referents, which falls within the definition of simulation (Gibbs, 2006). In embodied cognition outside of grounded cognition, simulation is equally important (Dijkstra & Post, 2015; Gallese, 2005). Even across the cognitive sciences, simulation is repeatedly emphasized: Predictive processing describes that offline internal models act as concept representations, which is simulation (Pezzulo, 2017; Pezzulo, D’Amato, et al., 2024; Williams, 2018). The field of spatial cognition is also increasingly demonstrating the importance of simulating spatial experience in abstract cognition (Bellmund et al., 2018; Buzsáki & Moser, 2013; Hartley et al., 2014). “Simulation theory” is the name of theories of cognition like Hesslow’s (2002, 2012; cf. also emulation theory by Grush, 2004), in physical reasoning (Battaglia et al., 2013; Ludwin-Peery et al., 2021), social cognition (Goldman, 1992; Shanton & Goldman, 2010), and in motor cognition (Jeannerod, 2001). These theories all have in common that they postulate the ability to activate mental states in absence of their sensory causes, and that this mechanism plays an important role in higher-level cognition. Based on this, we are confident in assuming that a core feature of representing concepts is the mechanism of simulation.

#### 4.1.2 Metaphoric Mapping

The mechanism of metaphoric mapping is similarly pervasive, although often implicitly, as it carries different names. It is defined as the sourcing of concrete representations to represent more abstract concepts (Jamrozik et al., 2016). A classic example of this is the representation of the abstract domains of emotion or valence in terms of concrete spatial directions, such as when someone is “feeling down” or their life is “on the up” (Casasanto & Bottini, 2014; Meier & Robinson, 2004). Despite doing significant theoretical legwork for a number of grounded cognition theories, metaphoric mapping is often not mentioned, and when mentioned it is not elaborated upon. For example, in his seminal perceptual symbol systems proposal, Barsalou (1999) explicitly stated that “metaphor most certainly plays a major role in elaborating and construing abstract concepts” (p. 600), but this is then not explained further. In other cases, the mechanism is described without being named as such: for example, Borghi et al. (2019) stated that the same directional movements are activated for concrete and abstract transfer (e.g., passing the pizza/passing the news), referring to Glenberg and Kaschak’s (2002) seminal ACE. The original findings and the attached theoretical explanations all rely on metaphoric mapping, constituting a metaphorical reuse of motor representations, yet it is not named so in either of the papers or most others discussing these (e.g., Glenberg & Gallese, 2012). Indeed, the action schema approach (Glenberg et al., 2008) is an instance of metaphoric mapping because it argues that the understanding of abstract relations relies on schemas that are grounded in or originate from action. Yet, it is most often viewed as distinct from conceptual metaphor theory and is rarely described as relying on metaphor.

These examples serve to demonstrate that metaphor use is pervasive in grounded cognition work (and its lack of recognition an additional instance of jangle), even if this recognition is not always explicit. Metaphoric mappings are an important and widely acknowledged feature of grounded cognition (Casasanto & Gijssels, 2015; Jamrozik et al., 2016). Further, beyond grounded cognition, metaphoric mapping is often equated with analogy (Gentner et al., 2001) or described as consisting of analogical mappings (e.g., Desai, 2022). Therefore, the expansive work on analogy in social cognition and communication (Landau et al., 2010, 2014; Thibodeau et al., 2019) and cognition generally (Holyoak, 2012; Indurkhya, 2013) also supports the importance of this cognitive mechanism. Indeed, one prominent author has even described analogy as “the very blue that fills the whole sky of cognition” (Hofstadter, 2001, p. 499). Based on its valuable contributions to grounded cognition, its success when disconnected from its theoretical baggage, and its universality, we also include metaphoric mapping in our minimalist grounded cognition account.

### 4.2. Background of the Minimalist Account

To arrive at the *minimalist account*, we inferred from prior work those parts that are 1) frequently used, within grounded cognition and beyond, and 2) natural kinds, i.e., constructs which exist independently of specific theoretical commitments (for full explanation, see below; Fried, 2017). This second constraint, of natural kinds, is a critical constraint to enable the kind of integration which we propose (see section 4.4 *Qualities of the Minimalist Account*). It is possible that there are other constructs that would meet our criteria, but we focused on these mechanisms because they are prominent in grounded cognition and allow integrating its productive research history. It is also necessary for the chosen mechanisms to originate from theory because this allows research applying these mechanisms to explain phenomena, as opposed to just describe them (Brick et al., 2022; van Rooij, 2019). We now justify each criterion in turn.

Firstly, demonstrating the frequency of these terms, a search in Google Scholar shows “embodied or grounded cognition” to be mentioned alongside “simulation” 230 000 times and alongside “metaphor” 544 000 times.^10^ This co-occurrence pattern demonstrates that the importance of these two mechanisms has been implicitly intuited by other researchers. This is similarly reflected in the way reviews of grounded cognition work treat theories, which often involves reducing these theories to their prominent mechanisms (cf. Table 1; examples are provided in section 3.2 on unsystematic empirical work). Therefore, we feel confident in assuming these to be two frequent and important aspects in grounded cognition. We used frequency as criterion because terms that are often mentioned in empirical grounded cognition work likely capture an important space of investigation (cf. Shapiro, 2019, who uses a similar approach to evince his “three themes of embodiment”). Further, those two concepts, which are extracted by many authors who characterize descriptive experimental work as important for their own experimental reasoning, are those which generate hypotheses, and which seem to raise important questions in the eyes of researchers. Therefore, choosing those mechanisms that empirical work has extracted is likely useful. Next, by focusing on mechanisms that were also used outside of grounded cognition in other research domains within the cognitive sciences, resulting theories are more likely to generate novel insights by ensuring commensurability, thereby generating integration. Finally, as demonstrated above, much independent work has arrived at the same two scientific concepts. This suggests that they capture something about the underlying nature of cognition (unless their frequent use has an alternative explanation).

Second, to ensure a solid epistemological foundation which is not speculative, the constructs we posit should be natural kinds, as opposed to nominal kinds. A natural kind is one which corresponds to the true structure of nature (e.g., *H_2_O*, because anything that does not have two hydrogen and one oxygen atom is no longer H_2_O), while a nominal kind is a category which captures superficial relations (e.g., *learning styles*, which ignores underlying factors; Fried, 2017; Schwartz, 1980). It is sometimes said that natural kinds “carve nature at its joints” because they are true categories that would exist whether humans would be alive to name them or not (Bird & Tobin, 2024). *Simulation* is a natural kind because its definition is based only on natural boundaries. If something does not consist of this description, in other words, if something is not a mental state re-activated in absence of the referent which usually activates it, then it is no longer simulation (Reilly et al., 2023). The explication of *metaphoric mapping* is similarly natural because it too is defined by what it does: simulating something more concrete in order to represent a (comparatively) more abstract concept. It is important that the features we choose here are tangible and real features as opposed to other, theoretical, constructs. By choosing such strict definition rules, we ensure that this account is epistemologically secure, as no matter what other theories turn out to be true or untrue, this account can remain. Furthermore, a second advantage of having natural kinds, is that it is more likely to align with other theories that also have natural kinds. While different theoretical frameworks can sometimes be incommensurable because they draw different boundaries into the causes of phenomena, this cannot be the case for natural kinds. Therefore, by these constraints, the minimalist account is able to gather from the significant prior work those components which can form a common and solid basis, thereby reducing conceptual clutter, and encouraging systematic empirical work.

### 4.3. Qualities of the Minimalist Account

The minimalist account addresses the issues described above because it is general in its postulates. With this generality, it gains certainty in its ability to capture the true underlying mechanisms. Further, it also does not make strong assumptions, because it only postulates those constructs and relations which already have very good evidence. This should in turn prevent theoretical enclaves, which will allow falsification instead of conceptual clutter and jingle-jangle, because all positions have been aligned. In short, the minimalist account trades potentially speculative sophistication for a humble and limited approach. It is an intentionally silhouetted view of grounded cognition, lacking in detail. In return for this lack of detail we gain something less measurable, but ever more important: A substantiated and common point of departure, which encourages future work to use this as a tool to meaningfully inform its falsificationist research and to progress incrementally.

The minimalist account, being an overarching theoretical framework, also improves the state of theorizing in grounded cognition more generally (Muthukrishna & Henrich, 2019), as a future research program should begin with the foundation of mechanisms which are well-established and well-understood (cf. Kitcher, 1995). Furthermore, it is in line with recent calls for theory reformation (Bringmann et al., 2022; Eronen & Bringmann, 2021), serving to remedy non-replication of results (Klein, 2014). Some foundational studies in grounded cognition have also been subject to non-replication (e.g., Colling et al., 2020; Morey et al., 2022), thus underlining the need for an overarching theoretical framework; in response to another non-replication it was recently proposed that ensuring successful replications “requires abandoning general theories […] and instead focusing on specific mechanisms” (Buckwalter & Friedman, 2024, p. 1). Finally, our approach does not just look to the past to solve problems, but also forward to solicit progress. It should encourage creative research thanks to its invitation for exploration (cf. Wegner, 1992), while nonetheless providing a unified framework which coordinates and connects research. One such prospective integration is the recent proposal regarding modal and amodal cognition by Kaup et al. (2024), which is similar in its minimalist spirit, preferring few, but substantiated and natural kinds in place of elaborate theories. The minimalist account and the mechanisms of simulation and metaphoric mapping fall fully within modal cognition. This example demonstrates the increased ease of integration when reducing theoretical elaboration.^11^ To this end, the minimalist account provides a simplified overarching theoretical framework which is necessarily limited in its detail but rather confident in its postulates, improving the current and prospective state of grounded cognition theorizing.

Beyond these strengths, the minimalist account also has limitations. It should be underlined that this framework cannot, by itself, solve all problems in the research field. Firstly, it is a theoretical framework, and not a theory in itself. This means that it cannot serve to explain many details. Instead, it serves to describe (explicitly) the components of knowledge currently available, on which most researchers are likely to agree. Further, we cannot purport that it solves problems in cognitive science beyond those which we have identified as most important above. For example, issues in non-replication can stem from the lack of a theoretical framework (Muthukrishna & Henrich, 2019), which we address; but it also comes from bad research practices, the complexity of the subject matter, and naïve use of statistical measures (Gernigon et al., 2024; Nosek et al., 2022; Open Science Collaboration, 2015). These practices causing ‘bad science’ relate also to structural issues in the field of science generally, like the preference for theory-guided over descriptive research in the reviewing of both manuscripts and funding applications, pressure to publish, and incentivizing the overselling of one’s research findings -- issues which are also prevalent in the field of grounded cognition (Zwaan, 2021). Lastly, our description of the proposed account does not provide specific recommendations for experimental practice. There are surely many ways, beyond theoretical reformation, to improve the state of empirical research in grounded cognition (see e.g., Ostarek & Huettig, 2019). In order to form a viable research program, all sides of the issue must be addressed, and the minimalist account addresses only a limited number.

### 4.4. Research under the Minimalist Account

The current synthesis remains intentionally agnostic about specific research practices. Of course, future grounded cognition research should adhere to the best-practice standards in science, applying principles of open science, such as pre-registration, rational sampling, and FAIR principles of data management (Appelbaum et al., 2018; Mayo-Wilson et al., 2021; Nosek et al., 2015), conducting adversarial collaborations (Logie, 2023) and performing replicable demonstrations across modalities, experimental designs, and populations (Fischer, 2024). Yet, beyond that we do not provide specific recommendations, aiming to ensure that the entirety of the proposal can be accepted unanimously by all grounded cognition researchers. Furthermore, specific recommendations would likely contain (potentially speculative) assumptions, and therefore have no place here. We emphasize that the minimalist account is a tool to improve the value of past and future research. It should be applied to enhance the ability of different studies to build on each other. We therefore make no suggestions on how to conduct research, merely to use the minimalist account as theoretical embedding (cf. Muthukrishna & Henrich, 2019). We similarly do not intend to formulate a novel research program, which is up to individual researchers to identify, based on their expertise. Nonetheless, we feel it necessary to describe in broad strokes the tone that future research can take with the minimalist account, and to flesh out our proposal with examples.

The minimalist account’s research program should depart from our simplified description of grounded cognition that is based on commonalities between theories, without the attached theoretical baggage in the form of unjustified details. Within this, and in agreement on the same basic principles, empirical research can depart in small steps, approaching a holistic description of concept grounding incrementally.

Research under the minimalist account should be less expansive and less assuming than much previous work. We do not suggest relinquishing all theoretical work. On the contrary, we argue that experimental work must align itself with a theory, but that in practice, research in grounded cognition has often exhibited poor theoretical allegiance, meaning it has not aligned with (any single) theory. Therefore, our minimalist account may, at first sight, seem less theoretical, but it is indeed more stringently theoretical than previous options. This relates to the same tension as the description above in which grounded cognition research is simultaneously framed as being too theory-laden (as opposed to descriptive) and engaging in too little theory-testing (being too discovery-oriented). Although, they sound similar, being theory-laden does not make research theory-testing. Indeed, the confirmatory, discovery-oriented research of past grounded cognition was theory-laden, but without actually attempting serious theory-testing via falsification. Being theory-laden prevents insights because it closes the research off from descriptive or exploratory research, and being discovery-oriented prevents these theories being refuted. By walking this thin line of theory-laden and discovery-oriented research, research in the field of grounded cognition has often managed to avoid paths towards more challenging but ultimately productive insights. Our solution, the minimalist account, does not assume detailed knowledge, which allows for a descriptive approach that is not closed off from discovering possible foundational principles. The issue of being closed off to new discoveries as a result of research being theory-laden is effectively reduced when giving empirical work the option of aligning with an unassuming and silhouetted theoretical framework. Beyond supporting descriptive research, future research should use the minimalist account to theory-test within the proposed framework of common assumptions. This was not possible before, where empirical work did not align itself with any theory at all, or when it did align, departed so far from another theory so as to make critical hypothesis testing impossible. Such benefits reflect our minimalist account’s focus on back-to-basics. Those explorative researchers who are less theoretically-minded can perform studies on “embodied effects”, without smudging over their theoretical allegiance, while others can pit opposing hypotheses against one another (but crucially, under the joint assumptions of the minimalist account; see also James’ distinction between knowing truth and avoiding error; James, 1897).

To give this proposal some shape, it may be valuable to provide two positive examples from past research which give an impression of what research under the minimalist account may entail. First, despite many detailed theories on metaphor, descriptive research such as the frequency of metaphor use in abstract concept representations, has been neglected. Determining such frequencies can be the basis of a strong-inference test: To what degree is metaphoric mapping involved in representing abstract concepts? Drawing on existing work, we can sketch how such an assessment could progress. The Lancaster sensorimotor norms (Lynott et al., 2020) map how strongly concepts draw on the various sensory (e.g., haptic or olfactory) or motor (e.g., foot or mouth movement) representations. The frequency of sensorimotor representations in abstract concepts can provide insight into the frequency of metaphor, with sensorimotor grounding of abstract concepts providing evidence in favor of metaphor. As an aside: The Lancaster sensorimotor norms only make such work possible because they are not theory-laden, consisting of basic, bottom-up constructs and natural kinds. The norms are therefore a product of the type of work which the minimalist account encourages, and this example demonstrates the compounding positive influences of the minimalist account.

A second demonstrative example of a minimalist research question is to determine the types of representations that can serve as grounding substrate. This question is rarely explicitly examined, despite forming an evidently important component of grounding concepts. We argue that this is the case because disputes over theoretical details and confirmatory research have taken precedence over descriptive and explorative research. It was recently pointed out that there is little a priori reason to exclude representations of physical invariants (unchanging features of the physical world like gravity or momentum) from these grounding substrates, given that they are richly represented (Friedrich et al., 2024). Indeed, recent experimental evidence building on this proposal has supported this inclusion (Friedrich et al., 2025). Yet it is only when divorced from their theoretical baggage, that the mechanisms in grounded cognition theories can account for this development. This demonstrates the importance of giving research the possibility to divorce from detailed theoretical descriptions. In order to arrive at a comprehensive understanding of concept grounding, the minimalist account should be the point of departure for future research because it does not presume to have a detailed account, and therefore allows for descriptive and explorative research that is close to nature, as opposed to theory.

## 5. Concluding Remarks

Grounded cognition made a daring leap in its early theories, which were innovative and influential far beyond the confines of grounded cognition. The field has grown from single lectures into one of the core pillars of a thriving research program. Despite the success of these theories in spawning much work, we argue that it is time to take a step back and re-evaluate how beneficial elaborate theories of grounded cognition are, and how much we trade epistemological security for apparent insight. Dogmatic adherence to historically influential theories, we believe, is no longer advancing our understanding of the nature of mental representations. Grounded cognition research, and much cognitive and psychological science, is missing bottom-up, descriptive, or exploratory research, and our minimalist account aims to stimulate such research. It takes grounded cognition research beyond discovery-oriented and theory-laden research that is based on speculative theories and plagued by unorganized constructs, to a serious field generating critical tests and valuable descriptions to stride ahead. We have tried to make evident here that the significant issues which impede grounded cognition can be readily relinquished. We presented the minimalist account, a simplified overarching grounded cognition framework based in the real, not theoretical world, which readily integrates with broader cognitive science and progresses beyond artificial borders drawn by previous theories. One should not confuse specificity with truth, and the minimalist account openly acknowledges its limits to gain credibility.

## Footnotes

For a seminal review, see Barsalou (2008), more recently Muraki et al. (2023) or a recent set of consensus papers (Banks et al., 2023; Bechtold et al., 2023; Ibáñez et al., 2023; Körner et al., 2023). For reviews focused on abstract concepts see Borghi et al. (2017) or Pecher et al. (2011); focused on neuroscience see Meteyard et al., (2012) or Martin (2016); focused on development (Wellsby & Pexman, 2014); for shortcomings Pecher (2018; Pecher & Zeelenberg, 2018); and for criticisms of the field see Goldinger et al. (2016), or Mahon and Caramazza (2008).

There is variation in the use of the term ‘embodied cognition’, with some positions being much more radical in their departure from standard cognitive science, such as radical embodied cognition, enactivism, the dynamical approach, ecological psychology, and others (Chemero, 2013; Gelder, 1998; Gibson, 1979; Hutto & Myin, 2012). There is likely little overlap in the contribution of this proposal and these, as they often reject the notion of mental representations, and instead emphasize interactions between person and environment, such as affordances. While we certainly endorse the embodied view of cognition, we do not position ourselves in these more radical camps and are more moderate alongside the authors we cite in the section on grounded cognition.

There is inexactness surrounding this terminology. The field we discuss in this paper, which emphasizes the importance of the modalities in concept representations, has been called “embodied cognition” (e.g., Glenberg et al., 2008; Lakoff, 2012), “grounded cognition” (e.g., Barsalou, 2008; Pecher, 2018), or “embodied and grounded cognition” (Borghi et al., 2019; Borghi & Pecher, 2011). Yet the term ‘embodied cognition’ refers to very many different fields that share few central tenets (Körner et al., 2016; Wilson, 2002). Therefore, we choose the term grounded cognition to 1) avoid unnecessarily limiting the modalities in which to ground concepts (cf. Friedrich et al., 2024), and 2) to specify that, within the many versions of embodied cognition, we focus on concept grounding (in line with Kiefer & Barsalou, 2013).

The term “tropism” was introduced by Myachykov et al. (2014) as replacement for the original term, to prevent confusing the general theoretical approach of grounded cognition with the specific evolutionary mechanism of “groundedness” proposed by Fischer (2012).

Please note that we have diverged from our previous naming convention for this theory, as evident in the table. In a prior paper we called this theory “simulation theory”, and now “perceptual symbol systems”. We choose this switch to distinguish better between the mechanism of simulation and the theory perceptual symbol systems.

We approach these issues from a metatheoretical perspective; for specific experimental recommendations see Ostarek & Bottini (2021) or Ostarek & Huettig (2019).

This is a much-simplified sketch of the process, we acknowledge that there are details and caveats to this approach. In this sense, the word ‘better’ refers to a full and complex evaluation of a given theory. Therefore, abiding Occam’s Razor, a simpler explanation would be preferred to a complicated one (cf. Lakatos, 1976). We acknowledge these and other caveats, but they are not further relevant for the current paper.

A similar process occurred around the automatic association of space and numbers. Original findings demonstrated that numbers are grounded in space, such that small numbers shift attention into the left hemispace and larger numbers shift attention into the right hemispace (Fischer et al., 2003). Following a failed replication (Colling et al., 2020), this claim about automatic attention shift was refined by considering individual differences in object counting and the degree to which the experiment demands processing depth (Shaki & Fischer, 2024). A further proposal argues that this and other spatial-numerical association findings depend on the format of representation, whether it is symbolic or non-symbolic, activating different pathways (Prado & Knops, 2024). Such proposals cannot integrate with specific grounded cognition theories, but can integrate with the minimalist account.

We are not the first to point this out, but seemingly the first who propose this as a viable unifying theoretical framework. As an example, it has been mentioned, almost as an aside, in Gallagher (2018, p. 357): “… the type of work done by […] Barsalou (1999), or by Lakoff and Johnson (1999), showing how, by simulation or metaphor, respectively, one can explain the embodied roots of abstract thought”.

The boolean searches for these statistics were *(“embodied cognition” OR “embodiment” OR “grounded cognition”) AND “simulation”* and *(“embodied cognition” OR “embodiment” OR “grounded cognition”) AND “metaphor”* respectively. The search was performed on Google Scholar on January 8, 2025.

Another example is work by Bub and Masson (Bub et al., 2008; Bub & Masson, 2006), which explored the content of simulations and whether it involved simulating merely passive action (e.g., holding an object without the purpose of acting with it) or acting with it. While this research is an excellent example of explorative research with few strong top-down assumptions, it has not been able to effectively inform theory, because there is no overarching theoretical framework. Therefore, it had to smudge over theoretical allegiances leading to an inability to be integrated with other work. We thank an anonymous reviewer for making us aware of the relevance of this line of research.
